# Interleukin 17-Mediated Host Defense against *Candida albicans*

**DOI:** 10.3390/pathogens4030606

**Published:** 2015-08-12

**Authors:** Florian Sparber, Salomé LeibundGut-Landmann

**Affiliations:** Section of Immunology, Institute of Virology, University of Zürich, Winterthurerstrasse 266a, Zürich, CH-8057, Switzerland; E-Mail: florian.sparber@uzh.ch

**Keywords:** interleukin 17, chronic mucocutaneous candidiasis, mouse models

## Abstract

*Candida albicans* is part of the normal microbiota in most healthy individuals. However, it can cause opportunistic infections if host defenses are breached, with symptoms ranging from superficial lesions to severe systemic disease. The study of rare congenital defects in patients with chronic mucocutaneous candidiasis led to the identification of interleukin-17 (IL-17) as a key factor in host defense against mucosal fungal infection. Experimental infections in mice confirmed the critical role of IL-17 in mucocutaneous immunity against *C. albicans*. Research on mouse models has also contributed importantly to our current understanding of the regulation of IL-17 production by different cellular sources and its effector functions in distinct tissues. In this review, we highlight recent findings on IL-17-mediated immunity against *C. albicans* in mouse and man.

## 1. Introduction

*Candida* spp. are frequently found in the human microflora. They colonize the oral, intestinal and vaginal mucosa, as well as the skin of healthy individuals [1]. They shape microbial communities in these compartments and interact with immune cells for the development and homeostasis of the immune system. However, many *Candida* spp. act also as opportunistic pathogens and can cause a wide range of fungal diseases [2]. Of all *Candida* species, *C. albicans* is among the most prevalent and most studied ones. It can cause superficial infections of the skin and the oral and vaginal mucosa [3], as well as more severe systemic infections, which are associated with a high degree of mortality and morbidity in those who survive [4,5]. The fungal pathogenicity depends on the host’s immune status, and disease symptoms arise primarily in immunocompromised individuals [6]; however, *C. albicans* can also cause disease in otherwise healthy individuals, such as is the case, for instance, for vulvovaginal candidiasis [7].

The conditions predisposing for candidemia are linked to modern medical treatments and include chemotherapy, immunosuppressive therapy, deep tissue surgery or the insertion of central venous catheters [8,9]. Mucocutaneous candidiasis, on the other hand, is a frequent complication in AIDS patients displaying low CD4^+^ T cell counts as a consequence of the viral infection and in severe combined immunodeficiency (SCID) patients with more general T cell defects [10]. More recently, congenital defects in genes linked to the interleukin 17 (IL-17) pathway have also been associated with mucocutaneous candidiasis [11]. Primary immunodeficiencies have thus emerged as an important risk factor for opportunistic (fungal) infections [12]. In this review, we focus on IL-17-mediated antifungal immunity and discuss the current understanding of the regulation of IL-17 production and the IL-17-mediated effector functions in response to *C. albicans*. The main focus is put on mucocutaneous *C. albicans* infections, but we also discuss the role of IL-17 in the context of vulvovaginal candidiasis (VVC) and systemic candidiasis.

## 2. IL-17 Family of Cytokines

The first gene of the IL-17 cytokine family was cloned in 1993 [13]. Since then, six IL-17 cytokines (IL-17 A to F) have been identified in human and mouse [14]. IL-17A and IL-17F share the highest amino acid sequence homology. They can form homo- and hetero-dimers, and they are often found to be co-expressed. Besides their protective role against *C. albicans*, IL-17A and IL-17F have gained much attention due to their capacity to promote autoinflammatory diseases, such as psoriasis and rheumatoid arthritis, and targeting the IL-17 pathway has become a promising approach in treating some of these conditions [15]. IL-17C is functionally related to IL-17A and IL-17F, while IL-17E (also called IL-25) is associated with type 2 immunity [14]. Other IL-17 family members remain ill-defined.

The family of IL-17 receptors comprises five structurally-related proteins (IL-17RA to IL-17RE) [14]. The biological activity of IL-17A, C, E and F depends on IL-17RA, which can pair with any of the other four receptor subunits. While IL-17RC plays a requisite role for the function of IL-17A and F homo- and hetero-dimers, IL-17C and IL-17E (IL-25) signal via complexes composed of IL-17RA and IL-17RE or IL-17RA and IL-17RB, respectively. The orphan receptor subunit IL-17RD was suggested to modulate IL-17A signaling [16].

In contrast to most other cytokines, which trigger JAK-STAT signaling cascades, IL-17 receptors function via a distinct pathway that depends on Act1 (also referred to as CIKS) and activates NF-κB and MAP kinases [14] for the induction of pro-inflammatory mediators. An alternative pathway that also involves MAP kinases affects target gene expression by controlling the stability of mRNA transcripts [14].

Due to these unique features and the sparse homology to other known cytokines or receptors, the IL-17 cytokine family is thought to represent a distinct ligand-receptor signaling system that has been well conserved throughout evolution. IL-17 homologs have been cloned and characterized from many different vertebrates [17,18,19,20,21,22,23,24], but also from invertebrates, such as sea quirt, sea urchin and mollusks [25,26,27].

In humans, rare mutations in the genes encoding IL-17F, IL-17RA, IL-17RC, RORc and Act1 are strongly associated with chronic mucocutaneous candidiasis (CMC) [28,29,30,31]. In addition, mutations in STAT3 and DOCK8 and gain-of-function mutations in STAT1, which result in the absence of IL-17-secreting Th17, also underlie CMC [32,33,34,35,36,37,38,39,40]. Moreover, defects in CARD9, an adaptor protein of the innate pathway coupling fungal recognition to Th17 induction, is also associated with an increased susceptibility to *Candida* [41,42,43,44]. In most cases, these patients are affected by isolated CMC. Susceptibility to such a narrow spectrum of infections is surprising and indicates that the IL-17 pathway and, in particular, IL-17A and IL-17F play a particular and non-redundant role in host defense against mucocutaneous infections with *C. albicans*. In line with the critical role of IL-17 in antifungal immunity, fungal evasion strategies have been proposed [45]. However, the mechanisms of IL-17-mediated protection remain unclear. Studies with experimental models of cutaneous and oropharyngeal *C. albicans* infections over the last few years have contributed to a better understanding of the regulation and function of IL-17 immunity against *C. albicans*. Besides *C. albicans*, non-albicans species of *Candida*, in particular *C. glabrata*, can also affect patients with CMC. However, the role of IL-17 immunity in host defense against this fungus remains largely unexplored. In the following, we will reflect on some of the recent advancements in the field, most of which have been gained from animal models.

## 3. Cellular Sources of IL-17 during CMC

CD4^+^ T cells are generally accepted as the primary source of IL-17A and IL-17F. *C. albicans*-responsive CD4^+^ T cells belonging preferentially to the Th17 subset are found in the circulation of healthy individuals that have been exposed to *C. albicans* [46,47]. Consistent with a protective role of these cells in antifungal immunity, defects in T cells and more specifically in Th17 cells are a predisposing factor for mucocutaneous candidiasis (see above). In experimentally-infected mice, *C. albicans*-specific Th17 cells develop in the draining lymph nodes within four to seven days after onset of infection [48,49]. A significant proportion of these cells recognizes a conserved antigenic epitope contained in the important fungal virulence factors agglutinin-like sequence (ALS) 1 and ALS3 [48]. A fraction of *C. albicans*-specific Th17 cells in humans also reacts towards the same epitope [48]. Differentiation of *C. albicans*-specific T cells into IL-17-secreting cells depends on C-type receptor signaling that acts through the tyrosine kinase Syk and the adaptor CARD9 and leads to the production of Th17-inducing cytokines, such as IL-6, IL-1β and IL-23, by antigen-presenting cells (APCs) [50,51]. Fungal determinants activating this pathway are thus critical for the T cell fate decision. However, tissue-specific factors of the host may also contribute, because in response to systemic infection, *C. albicans*-specific T cells differentiate primarily into IFN-γ-secreting Th1 and to a lesser degree into Th17 cells [52]. The tissue-specific regulation of T cell differentiation is likely related to the distinct distribution of different types of APCs in different tissues. Langerhans cells are a prominent DC population in the skin [53], and they were shown to drive the induction of the Th17 response during cutaneous candidiasis, while being dispensable for the T cell response to *C. albicans* in the oral mucosa, where they are rare (Trautwein-Weidner, Gladiator and LeibundGut-Landmann, unpublished). The DC compartments regulating Th cell priming during systemic candidiasis have not been investigated to our knowledge, but they are most likely distinct from those acting in barrier tissues.

Similar to the human situation, *C. albicans*-specific Th17 cells in infected mice form memory that remains stable over time, also after fungal elimination, and they mediate a slightly accelerated fungal clearance during re-infection via the cutaneous or the oral route [49,52]. In CD4-deficient mice, CD8^+^ T cells provide an alternative source of IL-17A in response to the fungus and functionally compensate the lack of Th17 cells [49], although these cells are no major players in this model if CD4^+^ T cells are present.

The experimental infection of mice with *C. albicans* is acute, and the fungus is usually controlled rapidly in the presence of an intact IL-17 pathway. This indicates that IL-17 acts before Th17 are primed and capable of providing the cytokine. Indeed, IL-17A and IL-17F expression in the infected tissue is induced rapidly after infection [54], suggesting that the relevant source of IL-17 for fungal control is produced by innate and not by adaptive cells. RAG-deficient mice lacking T and B cells show a delay in fungal control during oropharyngeal candidiasis (OPC) [54,55], suggesting that RAG-dependent cells contribute to the acute response to the fungus. IL-17 expression by TCRαβ^+^ or TCRγδ^+^ lymphocytes one day post-infection was recently demonstrated by means of a fate reporter mouse in which an eYFP reporter irreversibly marks all cells with a history of IL-17A promoter activity [55]. Although RAG-deficient animals have a delayed fungal clearance, they do eventually control the infection and regain their initial body weight [54]. However, depletion of IL-17A and IL-17F in RAG-deficient mice completely abolishes their ability to control the body weight and the fungal burden [54], providing evidence for a RAG-independent source of IL-17 cytokines during experimental OPC. Innate IL-17-producing cells in the oral mucosa of RAG-deficient animals have been identified as CD90^+^ cells, consistent with them being innate lymphoid cells (ILCs) [54]. No evidence has been found for neutrophils contributing to the IL-17 response during mucocutaneous candidiasis in mice [56], although these cells may respond to autocrine IL-17A in some situations, and they appear to act as an important source of IL-17A in other types of fungal infections [57].

The existence of at least three different cellular sources of innate IL-17 during OPC illustrates the robustness of the innate immune system. While the absence of individual subsets of IL-17 secreting cells, as is the case in TCRβ-/- or TCRδ-/- mice, has no impact on susceptibility [54,55], the lack of both TCRαβ^+^ and TCRγδ^+^ T cells (as in RAG-deficient mice) causes some delay in fungal control and finally the absence of all lymphocytes and ILCs, as in RAG-deficient animals depleted of CD90^+^ cells or in RAG-Il2rg-deficient mice, resulting in a complete disability to control the fungus, similarly to the situation of IL-17 pathway deficiency [54]. The relative contribution of individual IL-17 producing cell subsets may be context dependent. During cutaneous candidiasis, TCRγδ^+^ T cells in the skin may represent the dominant source of IL-17 [58,59], similar to the situation in the psoriatic skin [60].

Human data and results obtained from mouse models of OPC and cutaneous candidiasis suggest that IL-17A and IL-17F are the predominant IL-17 family of cytokines for the protection against *C. albicans* [29,31,54,61,62,63,64]. IL-17A and IL-17F are co-expressed by Th17 cells and innate IL-17 producers (Gladiator, Sparber and LeibundGut-Landmann, unpublished), and they act in a partially redundant manner [54]. In addition to IL-17A and IL-17F, the related cytokine IL-17C is also strongly induced during OPC [62,64], and consistent with the notion that IL-17C is produced by non-hematopoietic cells [65,66], the regulation of IL-17C secretion is RORγt independent in the oral mucosa [62]. However, IL-17C and the IL-17C-specific receptor subunit IL-17RE are dispensable for host defense against *C. albicans*, even in the absence of IL-17A and IL-17F [62], excluding redundancy between IL-17C and the functionally most closely-related IL-17 family of cytokines.

## 4. Mechanism of Action of IL-17 during CMC

Research on IL-17 biology performed in recent years focused primarily on the identification and characterization of IL-17A- and IL-17F-producing cells, while much less effort was put into addressing the functional consequences of IL-17 signaling in biologically-relevant systems. Early studies attributed neutrophils a key role in IL-17-mediated responses. Many IL-17 target genes are linked to neutrophil trafficking [67,68], and forced expression of IL-17A can result in massive neutrophilia, while disruption of the IL-17 pathway can cause defects in the neutrophil response [69,70,71,72,73,74]. IL-17 signaling is thought to target mainly the non-hematopoietic compartment and epithelial cells, as well as fibroblasts expressing relevant target genes [75,76].

Neutrophils play an essential role during acute mucosal defense against *C. albicans*, in particular by preventing fungal dissemination [62,77]. Genes involved in neutrophil mobilization and trafficking, such as *Csf3*, *Cxcl1* and *Cxcl5*, are induced during OPC in an IL-17RA-dependent manner [78]. Surprisingly, however, disruption of the IL-17 pathway does not affect the neutrophil response during OPC: neutrophil infiltration to the infected tissue, their localization in proximity to invading fungi and their candidacidal activity are not altered in IL-17RA- and IL-17RC-deficient mice [62], indicating that neutrophils act independently of IL-17 in the oral mucosa. Similar results were reported from an experimental model of psoriasis, where neutrophil recruitment to the skin was also uncoupled from the IL-17 pathway [76,79]. The link between IL-17 and neutrophils may thus strongly depend on the tissue environment, consistent with the notion that most situations, in which the IL-17 pathway was described to affect neutrophil recruitment, were observed in the context of pulmonary infections [69,70,71,72,73,74].

Another group of IL-17 target genes induced during OPC are antimicrobial peptides (AMPs), including S100A8 and S100A9 (which together form the heterodimeric complex calprotectin), β-defensin 3 and lipocalin 2 [78]. These agents were shown to display direct fungicidal activity through membrane depolarization and permeabilization [80] or to act by depriving the fungus of essential metal ions [81,82,83]. Besides their microbicidal activity, many AMPs exhibit additional functions by acting themselves as chemoattractants for a variety of innate immune cells or by stimulating other cells to secrete cytokines. In this context, β-defensin-1 was recently proposed to regulate IL-17A production during OPC and may thus act upstream of IL-17 [84]. Consistent with a critical role in mucosal host defense to *C. albicans*, β-defensin-1- and S100A9-deficient mice are impaired in fungal clearance [84]. However, their defect in controlling the fungus is not drastic (compared to the high susceptibility of IL-17RA- or IL-17RC-deficient mice), suggesting a considerable degree of redundancy between different AMPs. Interestingly, lipocalin 2 is fully dispensable for immunity to OPC, despite its very pronounced IL-17-dependent transcriptional regulation during infection [85]. Adding to the complexity of the biology of AMPs, many of them are expressed by different cell types that are differentially regulated during infection. IL-17-depdendent regulation of AMPs is restricted to the *de novo* synthesis in epithelial cells, while neutrophil-derived AMPs are preformed and released from granules or the cytoplasm upon neutrophil activation [62], in some instances in the context of NETosis [86]. Given the high degree of redundancy between different AMPs and their complex and differential regulation in multiple cell types, the relevant effector molecules mediating IL-17-dependent immunity to *C. albicans* still remain unclear. Besides antimicrobial molecules with direct fungicidal activities, IL-17-regulated factors controlling tissue regeneration and repair may also contribute.

In absence of a functional IL-17 pathway, experimental oral infection with *C. albicans* causes persistent colonization of the oral mucosa. The fungal burden remains high over time with fungal hyphae restricted to the epithelium without dissemination to deeper tissues or the circulation [62,78], indicating that some barrier functions are maintained in the absence of IL-17 signaling. Gradual reduction of the inflammatory response to *C. albicans* with/despite the persistence of high fungal loads [62] suggests that immune regulatory factors may come into play to control immunopathology and prevent overt inflammation in the infected tissue at the cost of preventing the clearance of the fungus. Future work is needed to identify the factors responsible for the equilibrium between fungal persistence and fungal control during OPC in IL-17-deficient hosts.

## 5. The Role of IL-17 in Vulvovaginal Candidiasis

Vulvovaginal candidiasis (VVC) is the most common manifestation of *C. albicans* infection, affecting ~75% of healthy women worldwide during their childbearing age [7]. Antibiotic treatment frequently increases vaginal *C. albicans* colonization and development of symptomatic VVC [87,88], suggesting that alterations in the vaginal microbiota may lead to the dysregulation of the antifungal resistance in the vaginal mucosa. However, the specific immune factors responsible for these changes are unknown, and it remains unclear whether IL-17 immunity is involved. CMC patients with mutations in IL-17F and other genes associated with the IL-17 pathway display symptoms of VVC [30,35,42,89] (in addition to oral candidiasis, as discussed above), but whether the disruption of the IL-17 pathway is a direct cause for VVC or rather an indirect effect due to the general increase in fungal colonization in these patients is unclear.

Data from animal models on the role of IL-17 during VVC remain sparse and are controversial. While one group reported that IL-17A and Th17 cells enhance protection [90], data from another group suggest that fungal control and the calprotectin- and neutrophil-mediated immunopathogenic inflammatory response during experimental VVC is independent of the IL-17 pathway [91]. Future research will be necessary to establish the mechanisms regulating fungal growth and immunopathogenesis during VVC in more detail.

## 6. The Role of IL-17 in Systemic Candidiasis

The very first link between IL-17 and fungal infections was provided when Huang *et al*. infected IL-17RA-deficient mice with *C. albicans* via the intravenous route and observed that they rapidly succumbed from infection and were unable to control the fungus in the infected kidney [92]. This was well before the discovery of Th17 cells and the first description of polymorphisms in IL-17-related genes associated with CMC. The critical role of the IL-17 pathway in host defense against systemic *C. albicans* infection was confirmed recently [64,93]. Importantly, the mechanism of IL-17-mediated protection against disseminated infection differs greatly from IL-17 immunity in barrier tissues [93]. Expression of IL-17A and IL-17F was only weakly induced, and antibody-mediated blockage of the cytokines or their receptor had only a mild effect on innate fungal control, pointing to a developmental disorder in IL-17RA-deficient animals that affects systemic protection from *C. albicans* infection. The defect was mapped to natural killer cells, and supplementing the dysfunctional NK cell compartment of IL-17RA-deficient mice with wild-type NK cells was sufficient to correct for the inability of the mice to control *C. albicans*. Antifungal defense of NK cells was found to act via a previously unrecognized mechanism: NK cell-derived granulocyte-macrophage colony-stimulating factor (GM-CSF), which is induced in response to Syk-dependent fungal recognition by CD11c^+^ DCs, promotes that antimicrobial activity of neutrophils [93,94].

The link between the IL-17 pathway and NK cells provides an unexpected mechanism of how IL-17A and IL-17 regulate protection from systemic infection with *C. albicans*. Given the broad and profound defect of IL-17RA- and IL-17RC-deficient NK cells in cytokine production and cytotoxicity [48], which resembles unlicensed NK cells, it can be speculated that its consequences reach beyond anti-*Candida* immunity, but may also be relevant for immunity against other fungi, as well as bacteria, viruses and possibly tumors. Future research will provide details about the molecular mechanism of IL-17-dependent NK cell function in mice and explore the relevance of these finding for NK cell-mediated immunity in humans.

## 7. Conclusions

Exciting discoveries of primary immunodeficiencies in CMC patients have enhanced our understanding of the pathophysiology of the disease and taught an important lesson about the physiological role of IL-17 in mucosal host protection. Previous studies have focused on the analysis of peripheral blood cells from these patients, but tissue-specific approaches will be necessary in the future to gain new insights into the IL-17-mediated immune control at different mucosal surfaces at the site of infection. However, such advances remain difficult given the small number of patients identified, many of which are children.

Much of our current knowledge on the cellular and molecular regulation of IL-17 and its protective mechanisms against *C. albicans* has been gained from experimental mouse models that provide a valuable tool for studying antifungal immunity *in vivo* and in a tissue-specific manner. To what extent these results obtained in model animals are comparable to the human situation remains to be determined. An important limitation of the mouse models lies in the fact that mice are *C. albicans*-naive. Infecting mice with laboratory strains of *C. albicans* via the oral, vaginal or cutaneous routes induces an acute infection that is rapidly cleared by the innate immune system in immunocompetent hosts. To evaluate the mechanism of IL-17-mediated defense during chronic infection as seen in patients with CMC, modified versions of the mouse models should be developed to better mimic the situation in humans. This, together with broader studies in patients, will be critical for gaining a more detailed mechanistic understanding of the protective principles that may eventually be targeted in the clinic for improving patient outcome.
